# Do internalising or externalising behaviours in adolescence mediate the child maltreatment‐alcohol substance use relationship?

**DOI:** 10.1111/add.70016

**Published:** 2025-03-02

**Authors:** Mike Trott, Claudia Bull, Jake Moses Najman, Dan Siskind, Urska Arnautovska, Steve Kisely

**Affiliations:** ^1^ Princess Alexandra Hospital Southside Clinical Unit, Greater Brisbane Clinical School, Medical School The University of Queensland Woolloongabba Australia; ^2^ Metro South Addiction and Mental Health Service Brisbane Australia; ^3^ Queensland Centre for Mental Health Research The University of Queensland Woolloongabba Australia; ^4^ ALIVE National Centre for Mental Health Research Translation Brisbane Australia; ^5^ School of Public Health The University of Queensland Herston Australia; ^6^ School of Social Sciences The University of Queensland St Lucia Australia; ^7^ Departments of Psychiatry, Community Health and Epidemiology Dalhousie University Halifax Canada

**Keywords:** adolescence, alcohol use disorder, child abuse, child maltreatment, externalising, substance use disorder

## Abstract

**Background and Aims:**

Child maltreatment (CM) is associated with several negative mental health outcomes in later life, including alcohol and substance use (AU and SU). Internalising (e.g. anxiety and depression) and externalising (e.g. delinquency and anti‐social behaviour) behaviours have also been associated with CM, and with AU and SU. This study measured whether externalising or internalising behaviours in adolescence mediate the relationship between agency‐reported CM and hospital admissions for AU or SU.

**Design, setting and participants:**

Observational study linking administrative health data from Queensland, Australia, to prospective birth cohort data comprising agency‐reported CM up to 14 years (exposure).

**Measurements:**

Externalising and internalising behaviours at 14 years measured using the Youth Self‐Report (mediator) and administratively linked inpatient hospital admissions for AU and SU from ages 25–39 (outcome).

**Findings:**

Adjusted causal mediation analyses (*n* = 5092) found that externalising behaviours statistically significantly mediated 31% (*P* = 0.007) of the CM AU relationship, and 22% of the CM SU relationship (*P* = 0.016). Internalising behaviours did not statistically significantly mediate between CM and either AU or SU.

**Conclusions:**

In Queensland, Australia, externalising behaviours appear to partially mediate the relationship between agency‐reported child maltreatment and hospital admissions for alcohol and substance use, while internalising behaviours do not.

## INTRODUCTION

Child maltreatment (CM) includes all forms of physical and/or emotional abuse, sexual abuse, neglect or negligent care, leading to actual or potential harm to a child's health, well‐being or self‐worth [1]. Most commonly, CM is measured using self‐reported measures [2]. Studies using contemporaneous notifications to statutory agencies are less common [3] and may represent different populations [4]. Linking data from child protection systems to administrative health datasets is an effective method for collecting information on the long‐term consequences of agency‐reported CM.

Exposure to CM has been consistently associated with negative health outcomes in later life, including alcohol and substance abuse. For example, a recent study from the Childhood Adversity and Lifetime Morbidity (CALM) study, an administratively linked dataset using the Mater‐University of Queensland Study of Pregnancy (MUSP) cohort study [5], reported that the odds of inpatient admissions were 2.9 times greater for alcohol use (AU) and 3.3 times higher for substance use (SU) where there was any previous agency‐reported CM [6]. These findings concur with previous MUSP results showing that exposure to CM was associated with 72% higher odds of cannabis dependence [7]. Similar results have been identified in reviews examining the association between exposure to CM and subsequent AU and SU [8, 9, 10]. As well as exposure to CM, externalising and internalising behaviours have also been reported as exposures associated with AU and SU outcomes.

### Externalising behaviours

Externalising behaviours include traits such as hyperactivity, delinquency and anti‐social behaviour [11]. Exposure to CM has been associated with subsequent adolescent externalising behaviours in previous MUSP studies. For individuals with any agency‐reported CM, the odds of externalising behaviours (defined as the 90th percentile) at 14 years of age were 2.16 higher compared to individuals with no CM history [12]. Regarding SU, MUSP studies have shown that adolescent externalising behaviours are associated with significantly greater odds of cannabis dependence at age 21 [7]. This partly concurs with the findings from other cohorts, although other studies show no associations. For example, Picoito *et al*. [13] found that high and moderate externalising behaviours at the age of 14 were highly associated with poly‐substance use and antisocial behaviours, whereas Papachristou *et al*. [14] reported no significant associations between externalising symptoms between ages 3 to 11 and subsequent drug use. Regarding AU, systematic reviews have reported consistent associations between externalising behaviours and AU, with some evidence of a dose–response relationship [15, 16], as well as evidence that ongoing externalising behaviours post‐adolescence also predict AU in later life [17]. A systematic review examining externalising behaviours as a mediating variable between childhood adversity and AU/SU reported mixed results. Although the majority of results found that externalising behaviours did mediate the childhood adversity—AU/SU pathway, the range of proportions mediated was large (14%–79%) [18]. There is also a possibility that genetics play a role in the CM‐externalising‐SU pathway, with Handley *et al*. [19] reporting that this relationship is only significant for individuals with 1 to 2 copies of the *FKBP5* gene.

### Internalising behaviours

Internalising behaviours include traits such as anxiety, depression, worry and social withdrawal [11]. Yet, whether internalising behaviours mediate the child adversity—AU and SU paths remains unclear [18]. Primary studies using the MUSP cohort have demonstrated positive associations between agency‐reported CM and internalising behaviours at the age of 14 [12, 20]. Regarding associations between internalising behaviours and SU, the MUSP cohort found that adolescent internalising behaviours exhibit a significant protective effect on cannabis dependence [7], which concurs with a similar international study [21], however, other international studies have shown no association [22]. Similarly, in the case of AU, the Australian CLIMATE schools study found that although internalising behaviours were associated with ‘risky’ alcohol behaviours (more than 5 standard drinks on one occasion), this association was largely explained by externalising behaviours [23].

In summary, although data from MUSP and other primary studies have demonstrated associations between CM and AU/SU, as well as between CM and internalising and externalising behaviours, systematic reviews have shown these relationships to be more consistent in externalising than for internalising pathways in studies [15, 16, 18]. What remains unclear is the presence and strength of a mediation effect between CM, internalising and externalising behaviours and hospital admissions for AU and SU (representative of more severe cases of AU and SU). Determining this pathway is important because it may inform interventions or screening programs as preventative measures. The aim of this study, therefore, was to examine the mediating effect that internalising and externalising behaviours in adolescence have on the association between CM, AU or SU in later life.

## METHODS

Ethical approval was granted by The University of Queensland Human Research Ethics Committee (HREC) (2022/HE001215) and the Metro South Health HREC (HREC/2022/QMS/83690). A pre‐trial protocol was also registered with the Australian and New Zealand Clinical Trials Registry (ACTRN12622000870752). Reporting of this study followed Strengthening the Reporting of Observational studies in Epidemiology (STROBE) guidance [24], the Reporting of Studies Conducted Using Observational Routinely Collected Health Data (RECORD) Statement [25] and the A Guideline for Reporting Mediation Analyses (AGReMA) statement [26], see Table S1 for full AGReMA checklist.

### Data collection and administrative linkage

**FIGURE 1:**
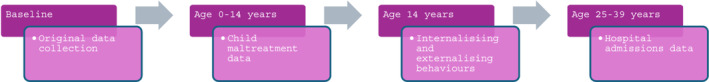
Timeline showing data collection and data availability dates.

This study used secondary data from the CALM study, the methodological processes of which have been explained in detail elsewhere [6, 27, 28]. In brief, in September 2000, notifications of CM to the Department of Families, Youth and Community Care (DFYCC) were anonymously linked to MUSP cohort records [29], providing statutory CM data for each person in the MUSP database. In 2023, the CALM study linked MUSP birth cohort (with 40 years of follow‐up) to Queensland‐wide administrative health datasets [27, 30], including hospital admissions from 1 January 2000 (the earliest date of data availability) through 1 January 2020, see Figure 1. The CALM study, therefore, included information from the children of all MUSP mothers, including up to 40 years of follow‐up information, CM data and inpatient admissions data. Information on the accuracy and quality of data linkage is available via the Queensland Data Linkage Framework [31].

### Materials and measures

CM was defined as any notification of any type of CM by any potential perpetrator to DFYCC from birth up the 14 years of age and was coded as a dichotomous variable. Although data for CM subtypes was collected, subtype stratification yielded sample sizes too small for robust analyses. At 14‐year follow‐up, mothers of the MUSP children were contacted and behavioural problems of the MUSP children were assessed using the internalising and externalising symptom subscales of the Youth Self Report (YSR) [32], see Figure 1. The YSR is a self‐report measure including a standardised checklist for adolescent behaviours, with internalising measures including anxiousness, depressive/withdrawn symptoms and somatic complaints, with externalising measures including aggression and rule‐breaking behaviours. The YSR has consistently yielded excellent psychometric properties [33, 34, 35]. In line with previous studies, internalising and externalising scores were dichotomised into the top 10% versus the remaining 90% to reflect symptoms of child psychopathology [32, 36].

Hospital admissions for AU were classified if participants had ever been admitted under the following International Statistical Classification of Diseases tenth revision (ICD‐10)‐AM codes (including subcodes): F10 (mental and behavioural disorders because of use of alcohol). Hospital admissions for SU were classified if participants had ever been admitted under the following ICD‐10‐AM codes (including subcodes): F11–F19 (mental and behavioural disorders because of other psychoactive substances use) [37]. Full information on specific codes included in the dataset can be found in Table S2.

### Statistical analysis

**FIGURE 2:**
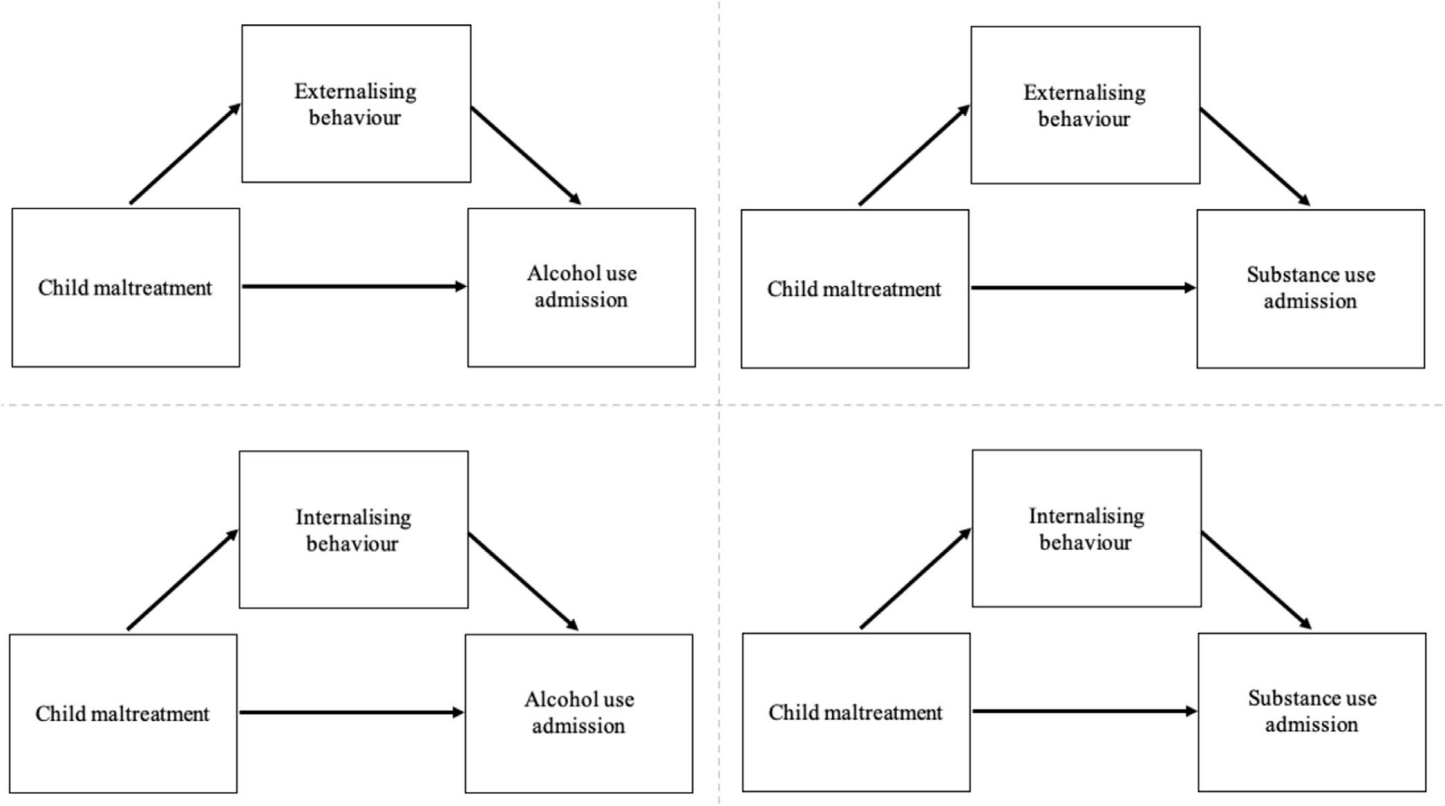
Mediation diagrams showing the tested mediations path for externalising (a) and (b) or internalising behaviour (c) and (d) in the child maltreatment and alcohol (a) and (c) or substance use disorder (b) and (d) relationship.

Causal mediation analyses was conducted using the ‘regmedint’ package in R [38, 39], testing the causal mediation models shown in Figure 2, with CIs determined using the Δ method [40]. No sample size calculation was conducted. Primary assumptions for the model included no unmeasured confounding variables, and temporal precedence between the exposure, mediator and outcome variables. CM and mediator interactions were also tested, and paths in the mediation model were adjusted for variables that have previously been independently associated with AU and SU in earlier CALM studies: baseline sex, maternal smoking, maternal binge drinking, maternal cannabis use and parental relationship status at birth [6]. As missing data were not missing completely at random (Little's test *P <* 0.001), all missing data were deleted listwise for primary analyses. However, sensitivity analyses were also conducted under the assumption of missing at random using predictive mean matching, with five imputed datasets generated and each imputation having a maximum of 50 iterations. Imputed datasets were analysed and pooled according to Rubin's rules [41]. Sensitivity analyses were also conducted to determine differences between included and excluded listwise groups, using internalising or externalising behaviours as continuous variables.

## RESULTS

Of the 7214 participants who originally took part in the MUSP study, 5092 participants (70.6%) completed the 14‐year follow‐up. All participants' administrative health data were linkable.

Of the 5092 participants, 51% were male at birth, 89% had mothers and fathers who were living together at time of birth, 29% were from low‐income households (defined as <AU$10400 per annum at time of birth, representative of the median household income at baseline) and 17% had mothers who did not complete high‐school level education (Table 1). Regarding CM, 8% of participants had any form of notified CM (mean age of notification 9.4). At 14 years of age, 469 children self‐reported internalising behaviours, whereas 523 self‐reported externalising behaviours. Between the ages of 25 to 39 years old, 1.6% of participants were admitted to hospital with AU, whereas 3.8% were admitted with SU. Attrition analyses found that First Nations Australians and participants whose parents were not living at the time of their birth were more likely to be excluded from the primary analyses because of missing data, see Table S3.

**TABLE 1:** Description of the MUSP cohort self‐reporting internalising and externalising behaviours at 14‐year follow‐up.

		Total sample *n* = 5097	Admissions for alcohol use (*n* = 81)	Admissions for substance use (*n* = 192)
Demographic information	Male sex at birth	51.3% (2611)	63.0% (51)	53.1% (102)
	First Nations Australian	4.5% (231)	11.1% (9)	6.8% (13)
	Family living together at birth	88.9% (4525)	86.4% (70)	82.8% (159)
	Low household income	28.5% (1453)	32.1% (26)	31.8% (61)
	Low maternal education	16.7% (852)	18.5% (15)	20.3% (39)
Child maltreatment	Any notification of child maltreatment	8.1% (413)	22.2% (18)	22.4% (43)
Behaviours at 14 y	Internalising behaviour	9.2% (469)	17.3% (14)	15.6% (30)
	Externalising behaviour	10.3% (523)	24.7% (20)	26.6% (51)
Hospital admissions at 25–39 y	Admission for alcohol use	1.6% (81)	–	–
	Admission for substance use	3.8% (192)	–	–

*Note*: All data is presented as percentage (*n*).

Abbreviation: MUSP, Mater‐University of Queensland Study of Pregnancy.

**FIGURE 3:**
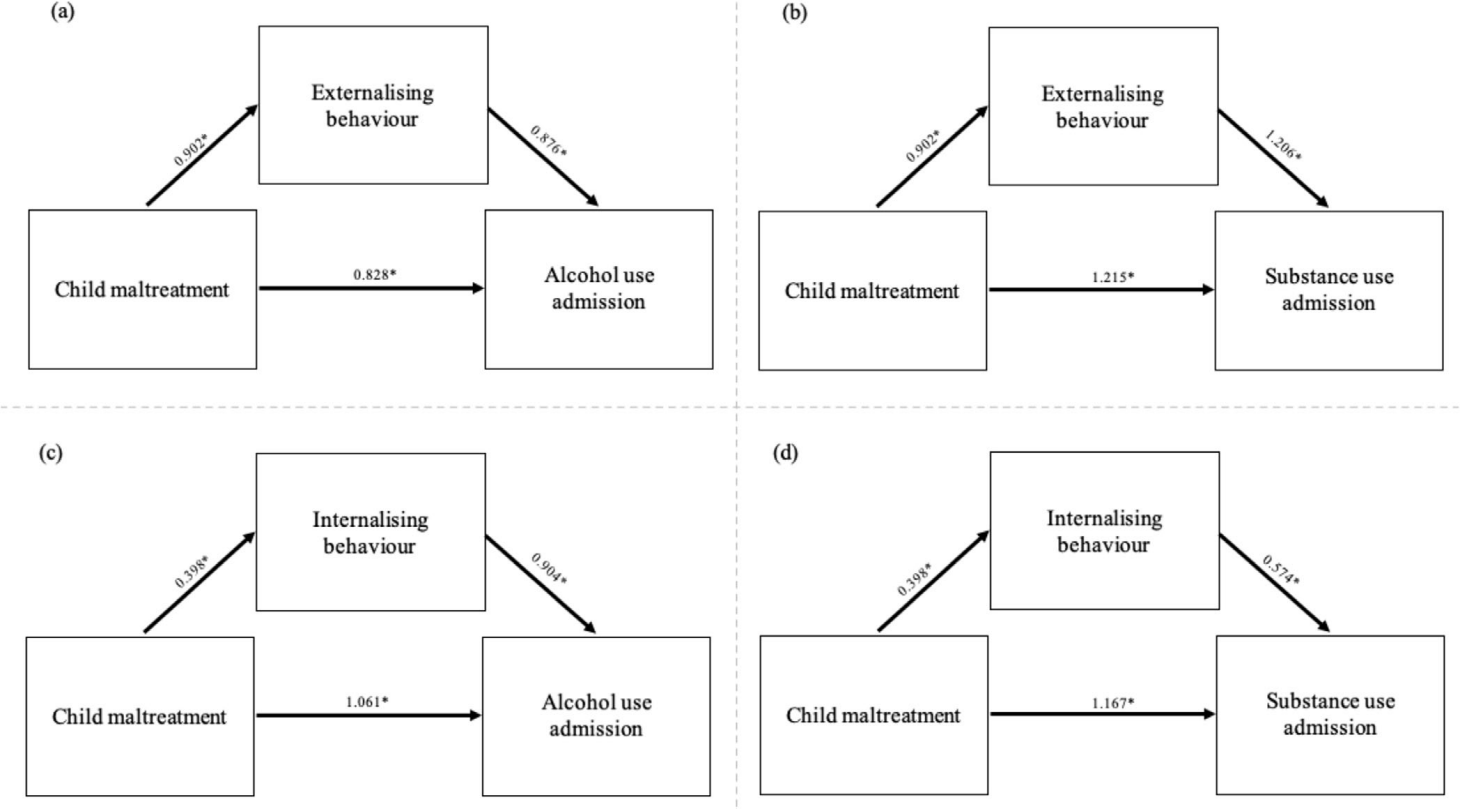
Mediation diagrams showing the mediation paths for externalising (a) and (b) or internalising behaviour (c) and (d) in the child maltreatment and alcohol (a) and (c) or substance use disorder (b) and (d) relationship.

Self‐reported externalising behaviours at 14 years of age significantly mediated 31% (*P* = 0.007) of the relationship between agency‐reported CM and AU admissions, whereas self‐reported internalising behaviours at the age of 14 did not significantly mediate this relationship (4.8%, *P* = 0.493). Self‐reported externalising behaviours at 14 years of age also significantly mediated 22% (*P* = 0.016) of the relationship between agency‐reported CM and SU admissions, whereas internalising behaviours at the age of 14 did not significantly mediate the relationship between agency‐reported CM and SU (5.4%, *P* = 0.292), see Table 2 for full mediation results, and Figure 3 for full path models. Sensitivity analyses conducted with the multiply imputed datasets and continuous mediator variables yielded no changes in the magnitude or significance of primary analyses, except that the proportion mediated in the AU—externalising—CM model was non‐significant (*P* = 0.12) when externalising behaviours was used as a continuous variable, see Tables S4 and S5.

**TABLE 2:** Causal mediation analysis of the mediating role of internalising and externalising behaviours at 14 years between child maltreatment and hospital admissions for alcohol use disorder and substance use disorder.

		Controlled direct effect		Pure natural direct effect		Total natural indirect effect		Total natural direct effect		Pure natural indirect effect		Total effect		Proportion mediated	
		β (95% CI)	*P*‐value	β (95% CI)	*P*‐value	β (95% CI)	*P*‐value	β (95% CI)	*P*‐value	β (95% CI)	*P*‐value	β (95% CI)	*P*‐value	Percent (95% CI)	*P*‐value
Alcohol use	Externalising behaviour	**1.06** **(0.09; 2.02)**	**0.031**	**0.94** **(0.33; 1.55)**	**0.003**	**0.24** **(0.02; 0.46)**	**0.033**	**0.99** **(0.26; 1.71)**	**0.007**	**0.19** **(0.05; 0.34)**	**0.045**	**1.18** **(0.50–1.86)**	**<0.001**	**31.0%** **(8.6–53.2%)**	**0.007**
	Internalising behaviour	0.78 (−0.55; 2.10)	0.251	1.01 (0.44; 1.58)	0.440	0.03 (−0.06; 0.12)	0.502	**0.99** **(0.41; 1.57)**	**<0.001**	0.05 (−0.03; 0.13)	0.223	**1.04** **(0.47; 1.61)**	**<0.001**	4.8% (0–18.6%)	0.493
Substance use	Externalising behaviour	**0.77** **(0.10; 1.44)**	**0.025**	**0.99** **(0.57; 1.42)**	**<0.001**	**0.17** **(0.00; 0.33)**	**0.045**	**0.90** **(0.40; 1.40)**	**<0.001**	**0.26** **(0.15; 0.37)**	**<0.001**	**1.16** **(0.68; 1.63)**	**<0.001**	**22.2%** **(4.1–40.3%)**	**0.016**
	Internalising behaviour	**1.32** **(0.48; 2.17)**	**0.002**	**1.19** **(0.83; 1.56)**	**<0.001**	0.04 (−0.03; 0.11)	0.309	**1.20** **(0.83; 1.58)**	**<0.001**	0.03 (−0.02; 0.08)	0.247	**1.23** **(0.86; 1.61)**	**<0.001**	5.4% (0–15.4%)	0.292

*Note*: Bold values indicate *P* = <0.05.

## DISCUSSION

This study examined the mediating effect of internalising and externalising behaviours on the association between CM and admissions for AU or SU in later life. The results illustrate that externalising behaviours at the age of 14 significantly mediated 31% of the relationship between CM and AU, and 22% of the relationship between CM and SU. However, internalising behaviours did not significantly mediate this relationship in this cohort.

These results are consistent with previous findings from the MUSP cohort, which found that externalising behaviours at the age of 14 were associated with a higher risk of cannabis dependence as at the age of 21. The current study extends these findings by examining the direct and mediating effect of externalising behaviours on subsequent AU and SU hospital admissions, which represent objective health service indicators. Although this is the first MUSP study to examine this specific relationship using objective outcomes, our results concur with previous mediation studies from other cohorts that used participant reported data. Handley *et al*. [19] found that externalising behaviour mediated the relationship between CM and adolescent (ages 15–18) marijuana dependence, although internalising behaviours showed no significant mediating effects. Oshri *et al*. [22] also reported that externalising behaviours in early adolescence mediated the association between CM and later adolescent (ages 13–15) marijuana dependence. The current study is also consistent with prospective studies that used participant reported data. For example, the CLIMATE schools study reported that although internalising behaviours were associated with risky alcohol consumption, this was largely explained in their models by externalising behaviours [23]. Interestingly, this association was only identified in men, and not women. Unfortunately, we were unable to stratify by sex because of small sample sizes.

Previous research (not in the context of CM) shows that externalising behaviours are well‐established predictors of substance use [42]. Conversely, internalising behaviours are less consistently associated. It may be that, much like externalising behaviours, the use of alcohol and other substances is an outward expression. When experiences of CM are added into the equation as a mediatory factor, the association between externalising and internalising behaviours and AU and SU becomes more ambiguous. This is because some forms of CM are explicit and tangible (physical abuse, sexual abuse), whereas other forms are less observable (emotional abuse, neglect). Therefore, possible mechanisms for our findings include potential maladaptive coping strategies (such as aggression and impulsivity) for the physical and emotional distress that is associated with CM, in turn leading to increased risk of AU or SU. Furthermore, CM has been reported to impair emotional processing and regulation, possibly leading to heightened impulsivity and risk‐taking behaviours in adolescence [41]. AU and SU are indeed risky behaviours. Conversely, it is possible that people with higher internalising behaviours during adolescence, following CM, may adopt different coping mechanisms, such as seeking solitary or lower‐risk activities (like gaming), than people with higher eternalising behaviours [42, 43]. Without the ability to examine mediation pathways between specific types of CM, internalising and externalising behaviours, and AU and SE, it is challenging to understand the nuance of these relationships. Therefore, future research with a sufficiently powered sample including information on CM subtypes is a critical area for investigation [43, 44].

Evidence from this study indicate that adolescent externalising behaviours could help quantify the risk of future hospital admissions for AU and SU in people who have a history of agency‐reported CM, as well as inform possible interventions. For instance, a RCT from the United Kingdom of cognitive behavioural therapy found that adolescents (mean age, 13.7) in the intervention group with higher levels of baseline externalising behaviours had lower instances of binge drinking at 2‐year follow‐up [45]. A further Australian RCT found that a targeted intervention of emotional education for adolescents with high levels of both internalising (anxiety sensitivity and negative thinking) and externalising symptoms (impulsivity and sensation seeking) yielded positive effects regarding binge drinking and hazardous alcohol use into early adulthood [46]. Interventions targeting young people with previous agency‐reported CM are warranted, as well as screening programs for adolescents with high levels of externalising behaviour, and longer follow‐up periods in interventional studies to determine long‐term effectiveness.

The results of this study should be considered within its limitations. First, the process and criteria for reporting CM in the 1980s and 1990s may not align with current reporting methods. Therefore, our use of agency‐reported CM is likely to be an underestimation of true CM prevalence, and subject to recording bias. Agency‐reported CM is also known to underestimate the true prevalence of CM as many cases go unnoticed and unreported. Second, our outcome measures (hospital admissions) may also be subject to recording bias and are likely to reflect the most severe cases of AU and SU. Our outcomes, therefore, should be considered as a conservative estimate. Third, externalising and internalising behaviours were self‐reported by MUSP cohort members at age 14. As such, it is unclear the extent to which these data accurately reflect pathological behaviours. Fourth, our results demonstrated only a partial mediation of externalising behaviours, suggesting that other unanalysed variables (such as gender and peer‐influences) may contribute to the casual relationship. Future research is warranted to explore CM gender interactions. Moreover, although the sensitivity analysis on multiply imputed data yielded results consistent with the primary analysis, the AU‐CM relationship was not significantly mediated by externalising behaviours when the mediator was treated as a continuous variable. This suggests that only extreme externalising behaviours mediate this relationship. Further research is warranted to confirm these findings in other cohorts. Last, because of the exploratory nature of this study, correction for multiple comparisons was less appropriate [47, 48, 49], and therefore, not conducted.

In conclusion, externalising behaviours partially mediated the relationship between agency‐reported CM and hospital admissions for AU and SU in the MUSP cohort, whereas internalising behaviours did not. These results provide new evidence to inform clinical guidelines regarding the need for early interventions in this priority population, especially in adolescents who have a history of CM. Future studies evaluating existing early interventions in this vulnerable population are warranted.

## AUTHOR CONTRIBUTIONS

**Mike Trott:** Conceptualization (equal); formal analysis (equal); investigation (equal); methodology (equal); writing—original draft (equal); writing—review and editing (equal). **Claudia Bull:** Conceptualization (equal); methodology (equal); writing—review and editing (equal). **Jake Moses Najman:** Conceptualization (equal); supervision (equal); writing—review and editing (equal). **Dan Siskind:** Conceptualization (equal); supervision (equal); writing—review and editing (equal). **Urska Arnautovska:** Conceptualization (equal); writing—review and editing (equal). **Steve Kisely:** Conceptualization (equal); data curation (equal); formal analysis (equal); methodology (equal); project administration (equal); supervision (equal); writing—original draft (equal); writing—review and editing (equal).

## DECLARATION OF INTEREST

None.

## Supporting information


**Table S1:** AGReMA Checklist.
**Table S2**: Full list and frequencies of ICD‐10 codes used to identify alcohol and substance use disorders.
**Table S3**: Attrition analyses results.
**Table S4**: Mediation analysis sensitivity based on multiple imputed datasets.
**Table S5**: Mediation analysis sensitivity analyses with internalising/externalising behaviours as continuous variables.

## Data Availability

Because of privacy, ethical and legal considerations, the administrative health data cannot be shared without direct approval from relevant data custodians and the Office of Research and Innovation of Queensland Health. Contact details for Queensland Health custodians can be found at https://www.health.qld.gov.au/__data/assets/pdf_file/0034/843199/data_custodian_list.pdf
